# Practical Use of Wearable Activity Measurement Devices in Orthopaedic Surgery: A Qualitative Analysis of Multidisciplinary Expert Experience

**DOI:** 10.3390/jcm15083009

**Published:** 2026-04-16

**Authors:** Dana Hazem, Emma Danielle Grellinger, Alex Youn, Seth Yarboro, Peter Richter, Sureshan Sivananthan, Bernd Grimm, Andrew Hanflik, WEARQ Group, Benedikt Braun, Meir Marmor

**Affiliations:** 1 Geisel School of Medicine at Dartmouth, Hanover, NH 03755, USA; dana.hazem.med@dartmouth.edu; 2 School of Medicine, University of California San Francisco, San Francisco, CA 94143, USA; emma.grellinger@ucsf.edu (E.D.G.); alex.youn@ucsf.edu (A.Y.); 3 Department of Orthopaedic Surgery, University of Virginia, Charlottesville, VA 22903, USA; seth.yarboro@gmail.com; 4 Department of Trauma and Orthopaedic Surgery, Klinikum Esslingen, 73730 Esslingen am Neckar, Germany; peter.h.richter@gmail.com; 5 ALTY Hospital Kuala Lumpur Malaysia, Kuala Lumpur 50450, Malaysia; drsureshsiva@gmail.com; 6 Luxembourg Institute of Health, 3555 Dudelange, Luxembourg; bernd.grimm@lih.lu; 7 Department of Orthopaedic Surgery, Southern California Permanente Medical Group, San Diego, CA 92123, USA; ahanflik@gmail.com; 8 Faculty of Medicine, University Hospital Tuebingen, Eberhard-Karls-University Tuebingen, 72076 Tuebingen, Germany; bbraun@bgu-tuebingen.de; 9 Department of Orthopaedic Surgery, University of California San Francisco, San Francisco, CA 94143, USA

**Keywords:** wearable sensors, orthopaedics, expert consensus, patient monitoring, implementation barriers, digital health, thematic analysis

## Abstract

**Background/Objectives:** Wearable activity monitors and sensor-based devices are increasingly used to quantify mobility, load, and recovery in orthopaedic patients, yet clinicians lack practical guidance on selection, implementation, and interpretation. This qualitative expert consensus study synthesized real-world experiences from leaders in orthopaedics, rehabilitation, biomechanics, and digital health who implemented wearables at scale. **Methods:** Semi-structured interviews were conducted with 16 experts (64% response rate) recruited via hybrid purposive and snowball sampling. Participants included orthopaedic surgeons and research scientists with 124 cumulative years of wearable experience across over 9000 monitored patients. Interviews addressed device selection, clinical workflow, data management, and adoption barriers. Data were charted into a structured extraction matrix and analyzed using Inductive Thematic Analysis and a Framework Approach, reported per COREQ guidelines. **Results:** Experts utilized diverse sensor platforms across arthroplasty, trauma, spine, and sports medicine. Four key themes emerged: (1) device selection prioritized usability and patient compliance over technical sophistication; (2) workflow required defined team roles to manage data volume and avoid clinical burden; (3) patient engagement favored simplified, actionable feedback amid divergent views on data transparency; (4) future outlook anticipated AI-driven proactive risk prediction. **Conclusions:** No single wearable suits all orthopaedic practices; success hinges on aligning sensor placement with clinical questions, rigorous data quality checks, and integration into care plans. This study offers a practical checklist and roadmap for point-of-care adoption.

## 1. Introduction

Wearable activity measurement (WAM) devices, including accelerometer-based activity monitors, inertial measurement units, pressure sensors, and camera-based systems, offer continuous, objective data on patient mobility and function in real-world settings [1,2]. In orthopaedics, these tools can quantify post-operative activity, weight-bearing, and gait quality, complementing traditional clinical examination and patient-reported outcome measures (PROMs) [3,4,5]. Systematic reviews have shown that WAMs can improve physical activity and support rehabilitation across diverse medical and musculoskeletal populations, yet most have focused on efficacy, technical performance, or single-condition cohorts rather than on how clinicians implement these technologies in busy orthopaedic services [6,7]. Qualitative work with clinicians and researchers has highlighted barriers such as data overload, workflow disruption, and unclear clinical utility, but these studies have rarely been orthopaedics-specific or drawn systematically on high-volume expert users across subspecialties [8,9]. Despite their promise, WAM technologies raise unresolved practical questions for clinicians, including what devices can measure, which metrics are most useful for specific orthopaedic problems, how to integrate sensors into peri-operative pathways, how to manage data and cost, and how to avoid common pitfalls. This study sought to bridge this gap by conducting in-depth interviews with experienced orthopaedic and musculoskeletal WAM users to generate an expert-informed, practice-oriented guide for selecting, deploying, and interpreting WAM-derived data in orthopaedic surgery and related care.

Recent advances in wearable activity measurement have accelerated adoption across multiple orthopaedic subspecialties. Miniaturized inertial measurement units (IMUs), high-capacity pressure insoles, and markerless computer vision platforms have expanded clinicians’ ability to quantify gait quality, weight-bearing adherence, and functional recovery in real-world environments outside the laboratory [1,2]. Validation studies have demonstrated moderate-to-strong agreement between consumer-grade wrist accelerometers and research-grade systems for step counting and sedentary behavior, and IMU-derived spatiotemporal gait parameters have shown acceptable reliability in post-arthroplasty populations [3,5]. Consumer-grade accelerometers have also demonstrated valid step counting in early post-operative orthopaedic patients using walking aids [10], and wearable technologies are increasingly reviewed as tools for supporting total joint replacement rehabilitation [11]. Machine learning applied to raw accelerometry signals can now classify activity types and detect fall risk with clinically actionable accuracy, opening the door to proactive, algorithm-driven patient monitoring [12,13]. Despite this technical progress, a persistent gap remains between device capability and routine clinical implementation: barriers including data overload, unclear reimbursement pathways, and workflow incompatibility continue to limit adoption [8,9,14]. Understanding how high-volume expert users have overcome these barriers is therefore essential to translating wearable technology from research settings into standard orthopaedic care. Wearable strategies in orthopaedics range from patient-owned consumer devices to dedicated clinical- and research-grade systems, and the challenges associated with each differ considerably. A complementary analysis by Braun et al. [15] focused on the patient-facing side of this equation, characterizing recruitment barriers in a bring-your-own-device (BYOD) orthopaedic trauma study. The present study addresses the clinician-facing side, examining how experienced practitioners select, deploy, and sustain wearable programs across a range of device types.

## 2. Materials and Methods

Design and Reporting: This study used in-depth, semi-structured interviews with experienced clinicians and researchers to understand how wearable devices are used in real-world orthopaedic care. Interview transcripts were reviewed systematically to identify common themes and recurring patterns in expert experience and recommendations. Reporting followed established standards for interview-based research (Consolidated Criteria for Reporting Qualitative Research—COREQ) [16]. A completed COREQ checklist is available in Supplementary File S1. The Diffusion of Innovations framework was used to guide discussion of how wearable technologies are introduced, integrated into practice, and maintained over time in orthopaedic care.

Expert Definition and Sampling: Participants were recruited via a hybrid strategy of purposive and snowball sampling. Potential participants were approached via email with a formal invitation outlining the study’s aims. Initial recruitment targeted recognized opinion leaders in orthopaedic surgery, rehabilitation, and digital health sciences; subsequent experts were identified through peer referrals at the conclusion of interviews. Of 25 potential participants approached via email, 16 agreed to be interviewed (response rate: 64%). The remaining nine candidates did not respond to the invitation. Inclusion criteria required experience with WAMs in a minimum of 100 patients or research participants. Data saturation was determined retrospectively during the analysis phase: upon reviewing the full set of transcripts, the research team confirmed that the final three consecutive interviews yielded no new thematic codes, indicating that thematic saturation had been reached. This approach is consistent with COREQ item 22, which requires that saturation be discussed, and is appropriate for Inductive Thematic Analysis where the complete dataset is reviewed before saturation can be formally confirmed.

Data Collection: The 25-item interview guide was developed by an international digital health committee (AO Foundation Smart Digital Solutions Task Force), iteratively refined through internal mock interviews to remove redundant or low-yield questions, and finalized by consensus. Domains included device and sensor types, clinical and research applications, patient eligibility, workflow and staffing, time requirements, costs and reimbursement, data security and ownership, outcome measures, obstacles, and future outlook.

The interviewers were trained in qualitative interviewing techniques prior to study commencement. Interviews were conducted by M.M. and B.J.B., both male, MD, orthopaedic surgeons. The interviewers had prior professional relationships with 2 of the 16 participants, facilitating open dialogue but requiring reflexive awareness of shared assumptions. To minimize bias, the interviewers adopted a neutral stance, utilizing a semi-structured guide to ensure consistency. Participants were informed that the study aimed to synthesize expert consensus for practical clinical guidelines.

Interviews were conducted via secure video conference using Zoom (Zoom Video Communications, Inc., San Jose, CA, USA) between February 2024 and October 2025. Interviews were conducted one-on-one, with no non-participants present. The semi-structured interview guide covered device selection, clinical workflow, and data management. Interviews lasted approximately 45–60 min and were video and audio-recorded and transcribed verbatim. Field notes were made during and immediately after each interview to capture non-verbal cues and contextual observations. Transcripts were returned to participants for comment and/or correction. No repeat interviews were carried out.

Data Analysis: Interviews were transcribed verbatim and analyzed using Inductive Thematic Analysis following the framework of Braun and Clarke [17].

Following transcription, data were systematically charted into a structured extraction matrix using Microsoft Excel (Microsoft Corporation, Redmond, WA, USA, 2024). This matrix was organized by participant (rows) and interview topic (columns) to facilitate a Framework Analysis approach. The structure distinguished between: (1) categorical variables (e.g., device types, patient volume) for descriptive quantification, and (2) verbatim narrative responses for inductive thematic coding.

Familiarization: Two researchers (D.H., [A.Y.]) independently read all transcripts and reviewed associated field notes to understand the depth of the data and ensure context was preserved during coding.

Coding: Initial codes were generated using Microsoft Excel. Coding was performed independently, followed by a consensus meeting to resolve discrepancies and merge overlapping codes (e.g., merging ‘patient burden’ and ‘compliance issues’).

Theme Development: Codes were grouped into higher-order themes representing the clinical workflow (Selection, Implementation, Data Actionability).

Member Checking: To ensure validity and resonance, the final manuscript—including synthesized tables, thematic maps, and interpretations—was distributed to all participants for review and approval as part of the group authorship process.

Trustworthiness: Credibility was supported by predefined expert criteria, a standardized interview guide, dual coding, and explicit linkage between raw answers, tables, and themes. Transferability was enhanced by detailed description of participant roles, practice settings, and patient populations. Dependability was addressed through an audit trail documenting coding decisions, table construction, and theme refinement within the spreadsheet. Confirmability was promoted through reflexive discussions about researcher assumptions and by grounding all interpretations in interview data and aggregated results. Member checking was performed at the transcript level to ensure data accuracy. Furthermore, participant validation extended to the thematic level; as group authors, the experts reviewed the final manuscript to verify that the overarching interpretations and conclusions accurately reflected their collective experience.

Outcome Measurements: Wearable devices used by expert participants measured a broad range of biometrics, summarized in Table 1. Consumer-grade accelerometers (ActiGraph, activPAL, Axivity Ax3) are among the most extensively validated platforms in orthopaedic and rehabilitation research. The ActiGraph has demonstrated high test–retest reliability (ICC > 0.90) for step count and activity intensity in ambulatory patients [5]. The activPAL thigh-worn monitor has shown excellent agreement with criterion measures for sit-to-stand transitions and ambulatory classification (sensitivity > 95%) in post-surgical populations. Insole pressure sensors, including the Novel Loadsol and Moticon platforms, provide valid measures of plantar force and weight-bearing symmetry (ICC 0.85–0.95) with adequate sensitivity to detect clinically meaningful changes during early fracture rehabilitation [13]. Multi-segment IMU arrays (APDM Mobility Monitor, Delsys) demonstrate acceptable concurrent validity against laboratory-grade motion capture systems for spatiotemporal gait parameters (ICC 0.80–0.95), though accuracy may decrease for complex movements or in populations with significant gait abnormality. Computer vision platforms, including Microsoft Kinect-derived systems, show moderate-to-good agreement with marker-based gait analysis for joint kinematics in clinical settings. Interpretation of wearable data in this study was primarily guided by expert consensus, with formal validity assessments referenced where available in the published literature.

Participant Characteristics: Specialties represented included orthopaedic surgery (*n* = 6; arthroplasty, sports medicine, trauma, foot and ankle), spine and neurosurgery (*n* = 1), physical medicine and rehabilitation (*n* = 1), basic science and biomechanics (*n* = 3), clinical research (*n* = 2), digital medicine and neurology (*n* = 1), and biomedical engineering/industry (*n* = 2). The majority of participants used wearables primarily for research (*n* = 11), with four combining research and clinical use, and one using wearables exclusively in clinical practice. Years of wearable experience ranged from 3 to 16 years (median 7.5 years; cumulative total 124 years), and the number of patients or subjects monitored per participant ranged from 100 to over 5000, with a collective total exceeding 9000.

## 3. Results

**Figure 1:**
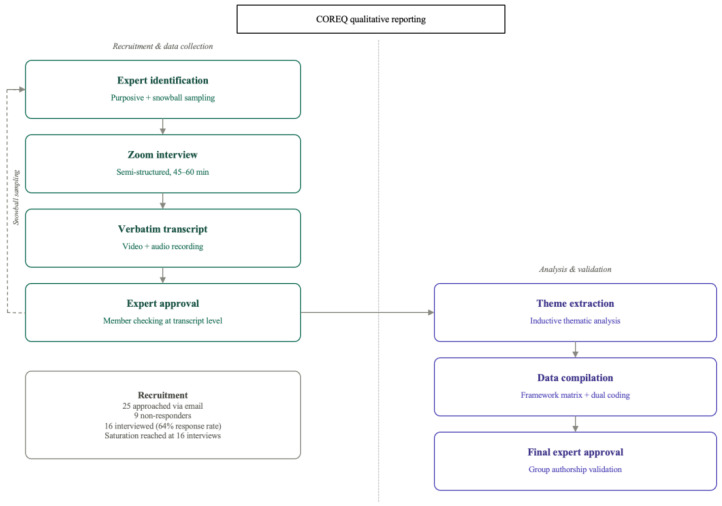
Participant flow diagram. Of 25 potential experts approached via email, 16 agreed to participate (64% response rate). Nine did not respond to the invitation. All 16 who enrolled completed the full interview and are included in the analysis. Reporting follows the COREQ checklist (Supplementary File S1).

Sixteen experts from North America, Europe, and Australia completed interviews, covering arthroplasty, trauma, spine, sports medicine, physiatry, biomechanics, digital medicine, and industry platform development. Collectively, they reported roughly 124 years of cumulative wearable experience and more than 9000 monitored patients or subjects. Recruitment ceased when additional interviews yielded no substantively new codes or categories, indicating thematic saturation, Figure 1 summarizes this process. 

### 3.1. Overview of Devices and Technologies

Experts reported using a broad spectrum of wearable systems, including consumer-grade wrist-worn trackers, hip- and ankle-worn accelerometers, shoe-based or insole pressure sensors, boot-embedded load monitors, multi-segment IMU arrays, and camera-based markerless motion capture. These devices measured step counts, time in different activity intensities, gait speed and spatiotemporal parameters, joint range of motion, ground reaction forces or surrogates, muscle activation, and occasionally heart rate and oxygen-saturation-derived metrics. Table 1 summarizes device families by primary function (e.g., activity tracking, gait and load assessment, segmental motion analysis) and associated biometrics, illustrating that no single platform met all needs across orthopaedic indications. Experts universally prioritized usability over technical sophistication, with one participant noting: ‘The ideal wearable is the one the patient already has… [biometrics] should come from their phone and not from a camera installed in their home’ (Participant 3). Another emphasized that practical design drives compliance: ‘For the lower extremity, the sock is ideal because a lot of patients don’t wear shoes at home’ (Participant 1).

**Table 1:** Devices and key details. Devices are grouped by primary function: (A) activity/step monitors, (B) gait and load assessment, (C) multi-segment motion analysis, (D) sport/performance monitors, (E) custom/research platforms.

Device/Platform	Duration on Subject/Patient	Data Read Frequency	Type of Device/Sensor	Key Metrics Measured
ActiGraph (Cat. A)	7 days	Once at the end	Wearable accelerometer-based activity monitor (IMU)	Steps, activity counts, intensity levels, sedentary time, posture, sleep metrics
Insole-based Google sensor (Cat. B)	One-time visit in the lab	Once after use	Insole sensor, IMU	Heart rate, HRV, blood oxygen, temp, steps, activity
Axivity Ax3 accelerometer (Cat. A)	7–14 days	Only at final follow-up	Triaxial accelerometer	Activity duration, intensity, and volume; movement frequency; posture and sedentary behavior
Computer vision (Kinect/camera) (Cat. E)	<15 min in clinic	Per session	Computer vision/camera	Kinematic/biomechanical tracking
Wireless insoles (Cat. B)	4–8 weeks	Once at the end	Insole pressure sensor	Pressure distribution, vertical ground reaction force
APDM Mobility Monitors (Cat. C)	2–6 min up to 3 h	Once after use	Multi-site IMUs	Gait/kinematics, EMG
BCI EmotiBit (Cat. C)	4–8 h (continuous same day)	Once after use	IMU, biosignals, EDA/GSR	Movement, biosignals, skin temp, EDA/GSR
Delsys EMG/IMU (Cat. C)	30 min–3 h	Once after use	EMG, IMU	Muscle activation, kinematics
Right Mechanics-Right Track (Cat. D)	6 months intermittently	Per follow-up visit	Custom device (motion, force)	ROM, gait/strength/coordination
Custom-developed Wayguard device (Cat. A)	4–6 weeks	Weekly	Triaxial accelerometer	Step count, mean gait velocity, step length
Pressure sensor in boot/cast (Cat. B)	2–6 weeks continuous	Once at the end	Pressure sensor, insole	Step count, cadence, force/load/weight
Shoe/hip-worn IMU sensors (Cat. A)	7 days	Once at the end	IMU	Gait speed, heel strike angle, heel strike impact, foot clearance, stride length, step variance
BioMotion Assessment Platform (Cat. E)	15 min, 3–4 times per episode of care	Per session	Custom platform (motion/muscle function)	Joint motion, muscle contraction
MOXY (Cat. D)	1 year	Per physical therapy visit	Near-infrared spectroscopy (NIRS) device	Skeletal muscle oxygen saturation
Zebra and Catapult (Cat. D)	Per practice/workout session for athletes	Daily or weekly	accelerometer and gyrometer	Top speed, acceleration, deceleration, rotational component
TracPatch (Cat. B)	4–6 weeks, 10–13 h/day	Daily or weekly	Adhesive IMU/smart patch	ROM, exercise, temp, steps, cadence, gait
Actibelt (Cat. A)	30 days	Once at the end	Triaxial accelerometer	Walking speed, cadence, stride length
activPAL (Cat. A)	7 days	Once at the end	Triaxial accelerometer	Steps, activity counts, intensity levels, sedentary time, posture, sleep metrics
Activ8 activity monitor (Cat. A)	7 days	Once at the end	Triaxial accelerometer	Steps, activity counts, intensity levels, sedentary behavior, sleep metrics

Notes: APDM Mobility Monitors: The duration of 2–6 min reflects a single standardized in-clinic gait assessment; the “up to 3 h” figure represents extended research protocol use and is not typical for routine clinical application. BCI EmotiBit: The duration of 4–8 h (continuous, same-day) reflects a full-day wear session for physiological monitoring and is distinct from the shorter assessment-based use pattern of the APDM. IMU = inertial measurement unit; EMG = electromyography; EDA/GSR = electrodermal activity/galvanic skin response; NIRS = near-infrared spectroscopy; ROM = range of motion; HRV = heart rate variability.

### 3.2. Clinical Applications and Use Cases

Wearables were used across the orthopaedic continuum, including early post-operative monitoring after fracture fixation or arthroplasty, outpatient rehabilitation, spine and sports medicine follow-up, and remote monitoring for patients living far from specialist centers. Experts emphasized two broad purposes: (1) capturing quantitative recovery trajectories (e.g., gait speed, load symmetry, light activity time) to complement PROMs and clinical examination, and (2) enabling proactive management, such as early identification of patients deviating from typical recovery patterns. Device choice and placement were consistently tailored to the clinical question: ankle or insole sensors for weight-bearing and gait after lower extremity surgery, trunk or multi-segment IMUs for complex motion analysis, and wrist devices primarily for overall activity rather than segment specific kinematics.

### 3.3. Implementation in the Clinical Workflow

Experts described small, defined teams responsible for wearable deployment, typically combining a lead clinician with research coordinators, physiotherapists, sports scientists or engineers and, in some programs, data analysts. Time requirements varied by system: simple activity trackers could be applied in minutes with batch data review, whereas multi-sensor or computer vision setups required dedicated assessments. Logistical hurdles often disrupted these workflows; as one researcher observed, ‘Sensors get lost in the mail when patients have to return them… sometimes there is no data on the accelerometer, although the battery is charged’ (Participant 2). Consequently, experts advised that ‘the best tools fit the patient’s existing habits’ to minimize burden (Participant 15).

Eligibility criteria generally required patients to ambulate independently or with minimal assistance, understand instructions, and have no conditions (e.g., severe cognitive impairment, certain psychiatric or eating disorders) that would make continuous monitoring unsafe or counterproductive. Table 2 summarizes common inclusion and exclusion patterns.

### 3.4. Practical Guidance for Device Selection and Data Quality

Experts converged on several principles for device selection: align the sensor location with the target joint or task; favor devices that are small, comfortable, and require minimal user actions; ensure adequate battery life for the intended monitoring window; and prioritize platforms that provide access to interpretable metrics and, when needed, raw data. Aesthetic appeal and discretion influenced adherence for wrist and ankle devices.

To secure data quality, programs used combinations of standardized application protocols, patient education, brief written or app-based instructions, daily or periodic checks for missing data, and validation against instrumented gait labs or established algorithms where available. Common pitfalls included device loss, misplacement, inadequate charging, erroneous classification of activities, and large unstructured datasets that strained local analytic capacity. Table 3 lists frequently reported technical issues, and the mitigation strategies experts found most effective.

### 3.5. Data Management, Privacy, and Feedback

Data were typically stored on secure institutional or HIPAA-compliant servers, with variable data ownership models depending on whether devices were consumer-grade, research-grade, or part of commercial platforms. Most experts regarded patients as the ultimate owners of their data, with de-identified datasets accessible to clinicians, researchers, or manufacturers under explicit consent. Approaches to patient feedback ranged from minimal reporting to routine sharing. While the consensus favored transparency to boost engagement (‘Patients want accountability… they want their progress to be measured,’ Participant 11), a minority view cautioned against real-time feedback. These experts argued that visibility could artificially alter recovery behaviors, noting that ‘participation in a clinical study alone changes behavior’ (Participant 9), potentially confounding outcomes.

### 3.6. Barriers, Solutions, and Outcomes to Track

The most commonly cited barriers were workflow burden for already stretched teams, lack of reimbursement and clear business models, uncertainty about which metrics truly matter for clinical decision-making, and variable clinician buy-in. Suggested solutions included integrating wearable assessments into existing clinic visits, delegating setup and monitoring to trained coordinators, focusing analyses on a small set of high-value metrics, and aligning programs with reimbursable remote monitoring codes or value-based care initiatives. Experts most frequently highlighted step counts, gait speed and spatiotemporal parameters, activity time and sedentary behavior, load or pressure profiles for protected weight-bearing, and PROMs such as KOOS, HOOS, EQ 5D, PROMIS, and SF 36 as core outcome domains. They emphasized that wearable metrics and PROMs are complementary rather than interchangeable, capturing performance and perception respectively. Table 4 maps commonly used biometrics to example orthopaedic questions, such as early detection of atypical recovery after arthroplasty or monitoring adherence to weight-bearing restrictions after fracture fixation. Across all 16 participants, compliance and patient engagement challenges were the most frequently cited barrier (13/16, 81%), followed by data quality and missing data issues (12/16, 75%), workflow integration and clinician buy-in difficulties (10/16, 63%), and reimbursement and cost barriers (9/16, 56%). Accelerometer-based activity monitors were the most commonly used device category, reported by more than 60% of participants, followed by insole and pressure sensors and multi-segment IMU arrays.

### 3.7. Lessons Learned and Future Directions

Across interviews, experts agreed that successful wearable programs start small, with a clear clinical question, a limited set of metrics, and a feasible team and workflow, then scale as value is demonstrated to clinicians, patients, and payers. Patient engagement depended on making the technology as unobtrusive as possible, giving feedback that patients could understand and use, and aligning monitoring with individual goals rather than generic step targets. Looking forward, participants anticipated tighter integration of wearables with electronic health records and an increasing reliance on AI for risk prediction. One expert predicted a fundamental shift in practice, stating: ‘In 3–5 years... 50% of medical decisions will be made on wearable data’ (Participant 9). However, achieving this requires solving the ‘data deluge’ and standardizing metrics; as another participant warned, ‘Raw data from wearables creates huge amounts of data… [which is] not meaningful unless you sample for greater than 5 min’ (Participant 5). This figure represents a single expert’s forward-looking prediction and is presented here as an illustrative opinion, not an evidence-based projection.

## 4. Discussion

This expert consensus study synthesizes practical, cross-disciplinary experience in using wearable devices in orthopaedic surgery and related musculoskeletal care. While the prior literature has largely focused on technical validation and single-cohort efficacy, our findings reinforce that there is no “one-size-fits-all” wearable solution for routine practice. Instead, the experts in this study suggest that successful implementation depends on a “question-first” approach—defining the clinical decision before selecting the device—and establishing rigorous workflow structures. The wearable landscape in orthopaedics spans a broad spectrum, from patient-owned consumer devices to dedicated research-grade and clinical-grade sensor systems, and challenges differ substantially across this spectrum. A complementary analysis by Braun et al. [15] examined recruitment characteristics in a bring-your-own-device study, in which patients used their own smartphones or fitness trackers to capture post-operative activity after orthopaedic trauma. That work identified older age, lack of device ownership, and limited technical literacy as the principal barriers to participation, obstacles that are largely specific to the BYOD model and less applicable to programs using dedicated devices issued and managed by clinical teams. The expert practitioners in the present study predominantly employed dedicated or research-grade platforms precisely to circumvent such barriers. Read together, the two studies illustrate that wearable implementation in orthopaedics presents distinct challenges depending on the device strategy adopted: BYOD approaches must contend with heterogeneous device ecosystems and patient-level technical barriers, while dedicated-device programs, as described by our experts, face different operational hurdles around workflow integration, data management, and clinician buy-in. A full understanding of the field requires attention to both ends of this spectrum.

Device Selection and Usability: Our first key finding indicates that usability and patient compliance consistently outweigh technical sophistication when selecting devices. This contrasts with much of the engineering-focused literature, such as the work by Keppler et al. [5], which prioritizes technical comparisons of sensor precision, or Dobkin [1], who emphasizes the granularity of motion capture. Our experts argued that high-fidelity data is useless if patients refuse to wear the device or if the form factor (e.g., cumbersome multi-segment sensors) disrupts daily life. This suggests that for clinical scalability, orthopaedic surgeons should favor “good enough” data from user-friendly consumer or hybrid devices over “perfect” data from research-grade systems that lack patient adherence. Furthermore, experts emphasized that this selection process is not intended to replace standard clinical tools. Instead, wearables and PROMs were described as “complementary rather than interchangeable”, with sensors providing the objective “performance” data (e.g., actual steps taken) that subjective “perception” scores often miss. This consensus mirrors the broader literature, where systematic reviews [3,4] and large prospective cohort studies [18,19] have consistently found only weak-to-moderate correlations between subjective scores and objective step counts, reinforcing that wearables capture a distinct aspect of recovery.

Workflow and Team Structure: Secondly, we identified that a defined team structure is critical to prevent “data deluge” and workflow disruption. This aligns with Azodo et al. [8], who identified workflow integration as a primary barrier to wearable adoption in broader healthcare settings. However, our results add specific granularity for orthopaedics: experts successfully mitigated burden by using small, specialized teams (e.g., a research coordinator and PT) rather than placing the onus on the surgeon. This refutes the notion often seen in early digital health studies that wearables can be “plug-and-play” tools for the solo clinician; without dedicated staffing for logistics and data filtration, clinical utility remains low.

Patient Engagement and Data Transparency: Regarding patient interaction, our experts reached a consensus moving toward simplified, actionable feedback, though opinions diverged on the risks of behavior modification. This supports the findings of Davergne et al. [7] and Latif et al. [6], whose systematic reviews demonstrated that activity trackers alone can significantly improve physical activity behaviors in musculoskeletal populations. However, our participants added a crucial caveat: real-time feedback can act as a confounder in research settings by artificially altering the recovery trajectory. Consequently, clinicians must distinguish between using wearables as a passive “measurement tool” (where feedback should be blinded) and an active “intervention” (where feedback is the mechanism of action).

Future Outlook and AI Integration: Finally, participants predicted a shift from reactive monitoring to proactive, AI-driven risk prediction. While current systematic reviews by Marmor et al. [4], Vogel et al. [20], and Small et al. [3] highlight that the field is still struggling to standardize basic metrics like step count and gait speed, our experts forecasted that within 3–5 years, wearable data could drive up to 50% of medical decisions. This optimism exceeds the current evidence base, suggesting a gap between expert vision and the current reality of unstructured, “noisy” datasets. Bridging this gap will require the standardization of data extraction and analysis protocols described in our findings.

Critical Appraisal and Broader Context: While our findings align well with the existing literature, several critical observations merit attention. First, the usability-over-precision preference endorsed by our experts contrasts with recent systematic reviews emphasizing that consumer-grade accelerometers significantly underestimate moderate-to-vigorous physical activity compared to research-grade systems, particularly in older post-surgical populations and in patients using gait aids during early post-operative recovery [5,10,21]. This suggests that clinical programs optimizing for compliance may inadvertently sacrifice data precision in exactly the patient demographic most in need of accurate recovery monitoring. Clinicians should therefore weigh the compliance advantages of consumer devices against the measurement limitations reported in the validation literature before committing to a specific platform. Second, the workflow structures described by our experts, typically small, dedicated teams of 1–5 staff, may not be generalizable to community hospitals or low-resource settings where research coordinators and data analysts are unavailable. Recent implementation studies have shown that wearable programs relying on dedicated coordinators frequently fail to scale beyond initial pilot cohorts, a limitation our participants acknowledged but did not fully resolve [8,14]. Third, while nearly all participants (15/16) used PROMs alongside wearables, the degree of integration varied substantially. Several experts noted clinically meaningful mismatches between objective wearable data and subjective PROM scores, patients reporting high function but low measured activity, or vice versa, consistent with published findings showing only weak-to-moderate correlations (r = 0.30–0.55) between wearable gait metrics and KOOS/HOOS scores in large arthroplasty cohorts [18,19]. These discordances, rather than being a methodological weakness, may represent genuinely distinct information domains, and future programs should be designed to capture and act on both rather than treating them as interchangeable. Finally, the anticipated AI-driven shift in clinical decision-making, while compelling as a future vision, must be contextualized: current AI applications to wearable data in orthopaedics remain largely in the validation and feasibility phase, with few prospective studies demonstrating that algorithm triggered interventions improve clinical outcomes beyond standard care [13,22].

Strengths and Limitations: Strengths of this work include the purposive sampling of high-volume wearable users across orthopaedic subspecialties and sectors, the structured capture of responses into analyzable tables, adherence to established qualitative reporting standards (COREQ) [16], and the validation of final themes by the expert participants. Limitations include a sample restricted to experts rather than typical community clinicians and a reliance on self-reported practices.

## 5. Conclusions

For orthopaedic clinicians considering wearables, this manuscript offers a pragmatic roadmap: start with a clear use case, pick devices that fit your patients and workflow, focus on a small set of meaningful metrics, and plan early for staffing, data management, and reimbursement. As technology and standards mature, integrating well-chosen wearable measures with PROMs and imaging has the potential to sharpen decision-making, individualize rehabilitation, and monitor outcomes at scale across orthopaedic populations.

### Clinical Significance

The practical framework synthesized in this study carries direct clinical significance for orthopaedic surgeons, physiatrists, and rehabilitation teams considering wearable implementation. By providing a structured, stepwise checklist grounded in collective expert experience across more than 9000 monitored patients, this work reduces the trial-and-error burden typically associated with first-time wearable adoption. Clinicians can use the device selection guidance (Table 1), inclusion and exclusion criteria (Table 2), and implementation checklist (Table A1) as actionable templates adaptable to their specific practice context, whether a high-volume academic arthroplasty program or a community-based trauma practice. The consistent finding that usability and patient compliance drive success more than technical precision provides reassurance that clinicians need not wait for perfect technology; well-chosen, familiar devices can generate clinically actionable data today. Furthermore, by framing wearables and PROMs as complementary rather than competing tools, this study supports a richer, more individualized approach to outcome monitoring that captures both objective performance and subjective patient experience.

## Figures and Tables

**Table 2:** Common inclusion and exclusion criteria.

Common Inclusion Criteria	Common Exclusion Criteria
Age/Population-Based: Adults (≥18 years), or specific focus groups (e.g., older adults, post-surgery populations, athletes, orthopaedic patients).	Medical Limitations: Non-ambulatory status, ICU/hospitalized patients, wound complications, comorbidities restricting required activities.
Diagnosis or Condition: Patients with specific diagnoses (e.g., ACL rupture, Achilles tendon rupture, ankle fractures).	Technological Barriers: Lack of required smartphone or support person for device management.
Functional Ability/Mobility: Ability to ambulate/move independently, weight-bearing status, healthy contralateral limb. Geography/Access: Resides within a set distance from the research center or has travel/access challenges.	Study Specifics: Study-specific exclusions such as intent not to return to activity or competitive sports, inability to follow up, or device-regulation-ineligible diagnosis.
Technology Access: Own (or access to) smartphone (sometimes limited to iPhone users) or ability to use/app interface.	

**Table 3:** Handling technical issues.

Technical Issue	Frequency	Solutions
Data transfer, storage, extraction, and missing/corrupt data	Very common	Frequent checks, standardized digital platforms, backup procedures, review algorithms for calibration, periodic manual review
Device calibration/setup complexity	Common	Clear patient/staff instructions, validated algorithms, repeat measurements, trained technical staff
Battery life, recharging, and power failures	Common	Preference for devices with long battery life, reminder systems, backup batteries, scheduled replacement
Hardware fragility and device loss or damage	Moderate	Durable device design, regular maintenance, tracking systems, clear usage guidelines
Data overload and challenges with analysis	Moderate	Data filtering and visualization tools, dedicated analysts or software for review, focusing on key metrics
IT infrastructure/compatibility with hospital/lab	Moderate	Use universally compatible hardware/software, IT staff collaboration, periodic system upgrades
App usability and connectivity issues	Occasional	Use patient-friendly apps, provide help desk/support team, offline modes for rural/remote areas
Patient or staff technical support needs	Occasional	Active support team or instructional videos, direct contact for troubleshooting

**Table 4:** Most useful biometrics.

Biometrics	Purposes and Typical Use Cases
Step Count	Monitoring general physical activity: used for motivating patient movement, remote monitoring, and progress tracking after procedures such as joint replacement and ankle fractures
Gait Speed	Assessing mobility recovery, particularly in trauma and post-surgical follow-up (e.g., after distal femur or knee surgery, and for longitudinal recovery monitoring)
Range of Motion	Tracking joint recovery and rehabilitation for knee, hip, shoulder, and ankle conditions; important for physical therapy outcome measurement
Activity Levels	Overall functional assessment in orthopaedics, monitoring sedentary time, moderate/vigorous activity, and daily counts for behavior change analysis
Heart Rate/HR Variability	General wellness tracking, stress monitoring, and correction for patient activation/engagement
Grip/Quad Strength	Recovery after upper/lower extremity injuries and surgeries, used to tailor physical therapy protocols
Cadence	Used for post-joint-replacement follow-up to distinguish quality of movement versus just frequency (useful when step count alone is insufficient)
Weight-Bearing	Tracking of load capacity, development and retention of prescribed weight-bearing adherence during (early) rehabilitation

## Data Availability

The datasets presented in this article are not readily available due to technical limitations. Requests to access the datasets should be directed to the corresponding author.

## Supplementary Materials

The following supporting information can be downloaded at: https://www.mdpi.com/article/10.3390/jcm15083009/s1, File S1: COREQ Guidelines. File S2: Group Authorship.

## Abbreviations

The following abbreviations are used in this manuscript:

AI	Artificial Intelligence
COREQ	Consolidated Criteria for Reporting Qualitative Research
EHR	Electronic Health Record
HRV	Heart Rate Variability
IMU	Inertial Measurement Unit
KOOS	Knee Injury and Osteoarthritis Outcome Score
PROMIS	Patient-Reported Outcomes Measurement Information System
PT	Physical Therapy/Therapist
TKA	Total Knee Arthroplasty
WAM	Wearable Activity Measurement

## Appendix A

**Table A1:** Stepwise checklist for launching wearable program in orthopaedic practice.

#	Step Name	Step Description
1	Define the clinical question and scope	Specify the primary decision or problem the wearable will inform (e.g., early detection of atypical recovery after TKA, monitoring adherence to protected weight-bearing after ankle fixation, remote monitoring for rural patients). Decide whether the initial scope is research-only, clinical-care only, or hybrid (e.g., quality-improvement pilot). Choose 1–3 priority outcomes you want to impact (e.g., readmissions, PROM improvement, rehab utilization, visit load).
2	Select target population and setting	Define inclusion and exclusion criteria: diagnosis/procedure, mobility level, age, language/cognitive status, technology access/comfort, and safety concerns. Decide where and when devices will be issued and retrieved (inpatient ward, pre-op visit, post-op clinic, PT, or remote mail-out/return). Start with one or two clearly defined pathways (e.g., primary TKA, ACL reconstruction) before expanding.
3	Choose device type and placement	Match sensor type and location to the clinical question: Activity and sedentary time → wrist, hip, or thigh accelerometer. Weight-bearing and load → insole/boot sensors or ankle/foot IMU. Detailed gait/joint kinematics → multi-segment IMUs or camera-based systems. Prioritize comfort, minimal daily patient actions, battery life that covers the intended monitoring window, and data access (summary metrics plus raw data if you need validation).
4	Build the team and workflow	Identify a clinical lead and a small operational team (e.g., research coordinator or nurse, PT/AT, and, if needed, data analyst or engineer). Map the workflow: Who applies the device, who educates the patient, who checks data, and when; how handoffs occur between inpatient and outpatient teams. Estimate time per patient for: device application, education, data download, and interpretation; confirm this is feasible within existing clinic or PT visit schedules.
5	Design the protocol and patient materials	Create a written protocol that covers: When devices are applied/removed. How long they are worn. Activity instructions and any safety restrictions. What happens if devices are lost, broken, or not charged. Develop simple patient-facing materials (1–2 pages or an app screen) with clear instructions, photos of correct placement, charging schedule, and contact information for problems. Decide what feedback patients will receive (e.g., steps per day, gait speed trend, simple “mobility score”) and how often.
6	Define metrics and data-quality checks	Select a small core set of metrics aligned with the clinical question (e.g., daily steps, gait speed, asymmetry index, light-activity minutes, time weight-bearing within prescribed range). Specify validity and quality rules: minimum wear time per day, number of valid days, acceptable ranges, and how to handle obvious artifacts. Establish routine data checks (e.g., weekly dashboards, flags for missing data or extreme outliers) and who is responsible for review and follow-up.
7	Plan data management, privacy, and ownership	Decide where data will live (institutional server, cloud platform) and who has access (clinicians, PTs, analysts, industry partners) under what permissions. Integrate consent language that clearly addresses what is collected, who can see it, how long it is stored, and whether de-identified data may be used for research. Clarify data ownership conventions (typically patient-owned with licensed clinical/research use) and ensure alignment with institutional policies.
8	Integrate into clinical decision-making	Define specific thresholds or patterns that trigger action (e.g., gait speed < X m/s at week 4 prompts PT re-evaluation; low step counts at 2 weeks post-op prompts outreach). Decide how wearable data will be displayed to clinicians (EHR note template, clinic dashboard, PDF report) and embed it in existing visit workflows. Train clinicians on how to interpret metrics and how to discuss them with patients.
9	Address reimbursement and sustainability	Map which activities might be billable (e.g., remote monitoring, extended care management) under current system codes. Estimate ongoing costs (devices, licenses, staff time, replacements) and build a simple business case referencing reduced visits, complications, or improved outcomes. Decide whether patients keep devices, return them, or use their own, and formalize replacement and loss policies.
10	Pilot, evaluate, and iterate	Start with a small pilot cohort and predefine success criteria (e.g., proportion of patients with ≥4 valid days, clinician use of data in notes, patient satisfaction, no major safety events). Collect structured feedback from patients and staff on usability, workload, and perceived value. Use pilot data to refine eligibility criteria, device choice, workflows, and metrics; then scale gradually to additional procedures or clinics.
