# Supplementary material for: Practical Use of Wearable Activity Measurement Devices in Orthopaedic Surgery: A Qualitative Analysis of Multidisciplinary Expert Experience
Source: J Clin Med. 2026 Apr 16;15(8):3009. doi: 10.3390/jcm15083009 (PMC13116219; doi:10.3390/jcm15083009)
Supplement: Supplementary file 1 [file jcm-15-03009-s001.zip › Supplementary File S2 WEARQ Group Membership.pdf]

Supplementary File S2
WEARQ Group Membership
The WEARQ (Wearables for Evaluation of Activity in Rehabilitation - Qualitative analysis) Group consists of the following members who participated as interview subjects and reviewed the final manuscript for accuracy:

1. Alexander Keppler, MD
   Department of Orthopaedics and Trauma Surgery, Musculoskeletal University Center Munich (MUM), LMU University Hospital, LMU Munich, Marchioninistr.
   15, 81377, Munich, Germany.
   Alexander.Keppler@med.uni-muenchen.de
2. Ali Boolani, PhD, FACSM
   Human Performance and Nutrition Research Institute, Oklahoma State University
   ali.boolani@okstate.edu
3. Amy M. Cizik, PhD, MPH
   Research Assistant Professor, Department of Orthopaedics, University of Utah
   amy.cizik@hsc.utah.edu
4. Andreas Brand, Dr.scient.med
   Institute for Biomechanics, BG Unfallklinik Murnau, Germany and Paracelsus Medical University Salzburg, Austria
   andreas.brand@bgu-murnau.de
5. Inger Mechlenburg
   Department of Orthopedic Surgery, Aarhus University Hospital, Denmark.
   Palle Juul-Jensens Boulevard 99, 8200 Aarhus N, Denmark
   inger.mechlenburg@clin.au.dk
6. Isabella Klöpfer-Krämer, Dr.phil
   Institute for Biomechanics, BG Unfallklinik Murnau, Germany and Paracelsus Medical University Salzburg, Austria
7. Jeannie F. Bailey, PhD.
   UCSF Orthopaedic Surgery
   Jeannie.Bailey@ucsf.edu
8. James E. Voos, M.D.
   University Hospitals Drusinsky Sports Medicine Institute, Cleveland, Ohio
   James.Voos@UHhospitals.org
9. Lorraine A.T. Boakye, MD
   Department of Orthopaedic Surgery, Perelman School of Medicine, University of Pennsylvania, Philadelphia, Pennsylvania, USA.
   Lorraine.Boakye@Pennmedicine.upenn.edu
10. Matthew Smuck, MD
    PM&R Division, Department of Orthopaedic Surgery, Stanford University
    msmuck@stanford.edu

11. Mischa Mühling, PhD
Institute for Biomechanics, BG Unfallklinik Murnau, Germany and Paracelsus Medical University Salzburg, Austria
12. Nan-Wei Gong, PhD
MIT Media Lab
nanwei@figur8tech.com
13. Prof. Ralph J. Mobbs MS, MD, FRACS
Professor of Neurosurgery, University of New South Wales, Sydney Australia
Chair, Wearables And Gait Research (WAGAR) Group, Sydney, Australia
ralph@drmobbs.com.au
14. Richard Bolander, Ph.D
TracPatch
rbolander@tracpatch.com
15. Stefano Alec Bini, M.D.
University of California San Francisco Department of Orthopaedic Surgery
Stefano.Bini@ucsf.edu
16. Victor H Hernandez, MD. MSc
Department of Orthopaedics, University of Miami.
Miami, FL. 33136
vhh1@med.miami.edu

All WEARQ Group members listed above:

- Participated in semi-structured interviews that formed the primary data for this study
- Reviewed interview transcripts for accuracy
- Reviewed the final manuscript, including synthesized tables, thematic interpretations, and conclusions, to verify that findings accurately reflected their collective experience
- Approved the final version for publication
